# Precision and accuracy of the dynamic endocast method for measuring volume changes in XROMM studies

**DOI:** 10.1242/jeb.249420

**Published:** 2025-02-19

**Authors:** Elska B. Kaczmarek, Ellen Y. Li, John G. Capano, Peter L. Falkingham, Stephen M. Gatesy, Elizabeth L. Brainerd, Ariel L. Camp

**Affiliations:** ^1^ Department of Ecology, Evolution, and Organismal Biology, Brown University, Providence, RI 02912, USA; ^2^ Department of Biological Sciences, Northern Arizona University, Flagstaff, AZ 86011, USA; ^3^ School of Biological and Environmental Sciences, Liverpool John Moores University, Liverpool L3 3AF, UK; ^4^ Department of Musculoskeletal and Ageing Science, Institute of Life Course and Medical Sciences, University of Liverpool, Liverpool L7 8TX, UK

**Keywords:** Biomechanics, 3D shape, Volume change, Musculoskeletal animation, Kinematics

## Abstract

The X-ray Reconstruction of Moving Morphology (XROMM) workflow enables precise and accurate measurement of the 3D skeletal kinematics underlying animal behaviors. The dynamic endocast method built upon that workflow to measure the rate of volume change within a bounded region of interest. We measured the precision and accuracy of the dynamic endocast method, using a fish oropharyngeal cavity as a case study. Despite overestimating instantaneous absolute volume, the endocast method was found to measure the rate of volume change with high accuracy. Importantly, it underestimated the rate of volume change, indicating that these measurements are conservative. We tested how variables such as alpha value and locator number impacted the accuracy of the endocast method. While the appropriate values for these variables are likely different for each application of the endocast method, we believe that our conclusion that the dynamic endocast method underestimates change in volume is generalizable.

## Teaser

**Summary:** The dynamic endocast method uses XROMM animations to measure volume change precisely and accurately, with a bias towards underestimating volume change, indicating that these measurements are conservative.

## INTRODUCTION

**Fig. 1.**
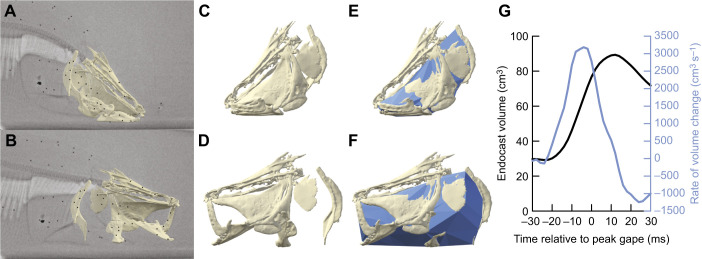
**The dynamic endocast method.** XROMM animation of *Chitala blanci* before (A,C,E) and during (B,D,F) suction expansion. (A,B) X-ray images with medial view of animated neurocranium, left-side bone meshes and surgically implanted bone and intramuscular markers (black circles). (C,D) Lateral view of animated bone meshes and (E,F) endocast. (G) Oropharyngeal endocast volume (black, left axis) and rate of volume change (blue, right axis) during a suction feeding strike. Modified from Li et al. (2022).

The ‘dynamic endocast’ method was developed for measuring change in oropharyngeal cavity volume in largemouth bass during suction feeding (Camp et al., 2015). Subsequently, it has been applied to studying oropharyngeal cavity volume during suction feeding in five other fishes (Camp et al., 2018, 2020; Gartner et al., 2022; Li et al., 2022; Whitlow et al., 2022), tidal volume during lung ventilation in sea turtles (J. G. Capano, L. N. Kim, C. J. Mayerl, J. Wyneken, R. W. Blob and E. L. Brainerd, unpublished), and oral cavity volume during food processing in macaques (Orsbon et al., 2020). To study a specific behavior, the dynamic endocast method requires an animation of the bones surrounding the volume of interest (Fig. 1A–D) which is used to create a bounded 3D shape (Fig. 1E,F). Endocast shapes are generated for each frame of an animation to produce time-varying measurements of instantaneous volume and rate of volume change (Fig. 1G; Camp et al., 2015).

Previous applications of the dynamic endocast method have used skeletal animations generated by X-ray Reconstruction of Moving Morphology (XROMM: Brainerd et al., 2010; Gatesy et al., 2010) to measure changes in oropharyngeal volume during suction feeding in fishes (Fig. 1). During this behavior, the bones surrounding the oropharyngeal cavity rotate and translate, producing rapid and forceful oropharyngeal expansion to suck in water and food. XROMM animations are generated by implanting each bone with radio-opaque markers, recording high-speed biplane videofluoroscopy of suction feeding, and tracking the markers in each X-ray video to calculate bone motions. These motions are applied to mesh models of each bone segmented from a computed tomography (CT) scan to create a precise and accurate animation (Brainerd et al., 2010). Dynamic endocasts are created from XROMM animations by placing virtual landmarks (i.e. locators) on the oropharyngeal surface of the bone meshes. These locators move with the bones, and their 3D coordinates define the shape of the endocast in each frame of the animation.

While the dynamic endocast method has been used previously, its precision and accuracy have not been measured. We hypothesize that the change in volume measured by the dynamic endocast method provides an unbiased measurement of the actual increase in oropharyngeal volume of the fish over time, while the absolute volume of each endocast is consistently biased towards an overestimate of water volume at every time step. We reason that this overestimate is caused by placing locators directly on bone surfaces, creating endocast volumes that include soft tissues and gill bars present within the oropharyngeal cavity of fishes. However, we predict that the change in volume should be unaffected because of the incompressible nature of these tissues and their consistent presence (Camp et al., 2015).

**Fig. 2.**
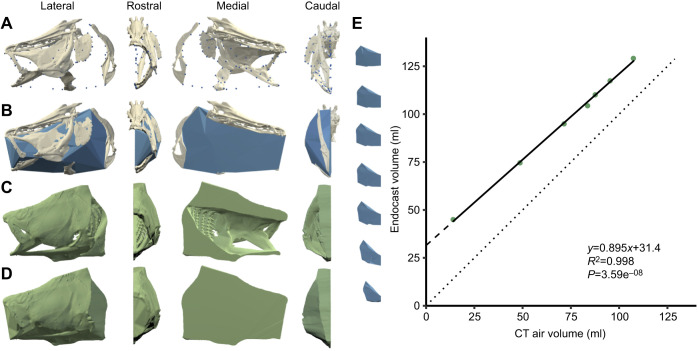
**Comparison of endocast volume and computed tomography (CT) air volume.** A CT scan of *C. blanci* is used to show the dynamic endocast method (A,B) and gold standard CT air volume method (C,D). (A,B) Pseudo-animated bone meshes, endocast locators (blue spheres) and alpha shape (blue). Locators on the midsagittal plane are not shown in the medial view in A. (C) The surface mesh and (D) wrapped mesh volumes were numerically subtracted to get the CT air volume. (E) The endocast and CT air volume (representing bilateral oropharyngeal volume) are plotted (green points). The solid line illustrates the line of best fit. The dotted line illustrates a 1:1 relationship. Alpha shapes (blue) for each CT scan are shown.

In this study, we measured the precision and accuracy of the dynamic endocast method's measurements of absolute volume and change in volume of the oropharyngeal cavity in a royal knifefish, *Chitala blanci*, and we assessed the effect of two variables in the volume calculations: the number of locators and the alpha value. The alpha value determines how tightly the endocast wraps around the locators. We created proxies representing cranial expansion during suction feeding by CT scanning a dead knifefish specimen in seven poses from fully closed to fully expanded, with air filling the oropharyngeal cavity (Fig. S1). To generate endocasts from these CT scans, we created a pseudo-dynamic XROMM animation and applied locators to the bone meshes. To create a gold standard for comparison, we segmented the volume of air in the oropharyngeal cavity in each CT scan (Fig. 2C,D). We determined precision and accuracy by comparing the oropharyngeal volume measurements of the endocasts with the air volumes in the CT scans (Fig. 2E). Additionally, we provide a detailed methodology for the dynamic endocast method and guidelines for validating the dynamic endocast method for other applications.

## MATERIALS AND METHODS

We begin with instructions for the dynamic endocast method and then describe our methods for measuring precision and accuracy in this study. These instructions assume marker-based XROMM (Brainerd et al., 2010) but would work just as well with markerless XROMM (Gatesy et al., 2010; Miranda et al., 2011).

### The dynamic endocast method

The steps for producing dynamic endocasts and measuring instantaneous rate of change in volume from marker-based XROMM animations are as follows. A tutorial for the dynamic endocast method is provided in the Supplemental Materials and Methods. (1) Collect XROMM data. (2) Import mesh models of the bones into an animation platform. (3) Attach virtual landmarks (locators) to the bones. (4) Animate the bones and their attached locators. (5) Export the 3D coordinates of the locators in each frame. (6) Calculate alpha shapes (and their volumes) using the locator coordinates. (7) Visualize the endocast alpha shapes along with the animated bones and improve fit of endocasts as needed. (8) Calculate the endocast volume change per time step.

Many of the steps above can be completed with various software; however, we will describe how we used Autodesk Maya (2018; Autodesk, San Rafael, CA, USA) and MATLAB (R2020a; The MathWorks, Natick, MA, USA), as well as custom XROMM Maya Tools (XMT; developed by David Baier and S.M.G., available at https://bitbucket.org/xromm/xromm_mayatools/) and custom-written MATLAB and MEL scripts (developed by A.L.C., P.L.F. and S.M.G., available at https://bitbucket.org/ArielCamp/dynamicendocast) for our pipeline.

Prior to using the dynamic endocast method, data for the XROMM workflow must be collected. This includes implanting markers into the bones of interest, recording high-speed biplane fluoroscopy videos of the behavior of interest, tracking the markers in the videos and calculating the rigid body transformations, collecting a CT scan of the bones, and segmenting each bone of interest in the CT scan (see Brainerd et al., 2010; Gatesy et al., 2010; https://gitbook.brown.edu/xromm/). Given the difficulty of producing XROMM animations, researchers generally animate the bones surrounding only half of the volume of interest and double that volume if bilateral symmetry is a reasonable assumption.

Once the necessary XROMM data have been collected, we import bone mesh models into Maya using XMT ‘imp’. Then, we place virtual landmarks (called locators in Maya) onto the surfaces of the bones, so as to effectively capture the boundaries of the volume of interest. When measuring unilateral volume, we create a plane in Maya (native Maya function), align it to the midsagittal plane of the animal and place locators on the plane. We parent the locators and the midsagittal plane to their respective bones (native Maya function) so that the locators inherit the bone's transformations upon animation. To animate the bones, we import rigid body transformations and apply them to their respective bones using XMT ‘imp’. Then, we export the world-space *xyz* coordinates of each locator using XMT ‘exp’ or the custom-written MEL script ‘exportTransWS’.

We use a custom-written MATLAB script to generate the endocast alpha shapes. Firstly, we import the locators' *xyz* coordinates into MATLAB. Then, we use the native MATLAB function ‘alphaShape’ to generate an alpha shape and calculate the alpha shape's volume for each frame. Lastly, we export the alpha shapes as mesh models (i.e. endocasts). It is important to note that the ‘alphaShape’ function requires a parameter (called ‘alpha value’) to be specified, determining how tightly the bounding alpha shape wraps around the point cloud.

A critical step in the dynamic endocast method is confirming that the alpha shapes correctly capture the volume of interest. To visually confirm this, we import the alpha shapes as mesh models into the XROMM animation in Maya using a custom-written MEL script. If the alpha shapes do not capture all of the volume of interest, it may be necessary to increase the number of locators or increase the alpha value. If the alpha shapes interpenetrate the bones, it may be necessary to decrease the alpha value or increase the number of locators. Further information on the effects of the number of locators and alpha value is provided in Results and Discussion, as well as in the tutorial provided in the Supplementary Materials and Methods.

Once the alpha shapes accurately capture the morphology of the volume of interest, their volumes can be analyzed. We filter the volume versus time data to reduce noise before calculating volume change (Δ*V*/d*t*) because calculating Δ*V* is effectively taking the derivative of the volume versus time plot, which amplifies noise.

### Measuring precision and accuracy of the dynamic endocast method

We measured the precision and accuracy of the dynamic endocast method using, as a test case, the change in volume of the oropharyngeal cavity of a dead royal knifefish specimen, *Chitala blanci* (d'Aubenton 1965). The methods we used for this validation study are described below. More details are provided in the Supplementary Materials and Methods.

These methods assess the accuracy and precision of the dynamic endocast method in two recent studies that also utilized alpha shapes to measure change in oropharyngeal volume during suction feeding (Camp et al., 2020; Li et al., 2022). However, earlier applications of the endocast method used the locators on the bones to control the movement of the vertices of a deforming polygon, rather than generating alpha shapes (Camp et al., 2015, 2018). While thoroughly assessing the precision and accuracy of the deforming polygon method is outside the scope of this study, we have included preliminary calculations of oropharyngeal volume using a deforming polygon (see Supplementary Materials and Methods).

To measure precision and accuracy of the (alpha shape) endocast method, we performed the following steps: (1) CT scanned the specimen in poses with varying oropharyngeal expansion; (2) measured oropharyngeal volumes using the dynamic endocast method; (3) measured oropharyngeal volumes using a ‘gold standard’ method; (4) calculated the accuracy and precision of the endocast method relative to the gold standard method; and (5) visualized the endocasts relative to the meshes produced using the gold standard method.

We manipulated the specimen in air to approximately simulate the cranial expansion that occurs in water during suction feeding. We clamped the body of the specimen in an upright posture and pulled dorso-caudally on the neurocranium (Fig. S1). Elevating the neurocranium caused depression of the lower jaw and hyoid and abduction of the suspensorium and operculum, mimicking the expansion that occurs during suction feeding. We CT scanned the specimen, which had radio-opaque markers implanted in the cranial bones of interest, at a range of positions between fully closed and fully expanded, and selected seven scans that best represented the full range of expansion. Each scan represented a time step in a pseudo time series.

First, we measured the volume of the oropharyngeal cavity in the CT scans indirectly using the dynamic endocast method. Because previous applications of the endocast method for studying suction feeding only animate one half of the head, we created endocasts of the left side of the oropharyngeal cavity. To create the endocasts, we segmented mesh models of the bones from one CT scan, obtained the *xyz* coordinates of the radio-opaque markers from each of the other CT scans, and used those *xyz* coordinates to calculate the rigid body transforms (RBTs) of the bones in each scan. We attached endocast locators to the bones in Maya and applied the RBTs to the bones to animate them. Then, we exported the *xyz* coordinates of the locators and used them to generate alpha shapes (i.e. endocasts) and calculate alpha shape volumes in MATLAB (Fig. 2A,B).

We repeated this method multiple times while varying the number of locators placed in Maya and the alpha value used to generate the alpha shapes in MATLAB. We used eight sets of locators, where the number of locators in each set varied from 21 to 216, increasing by 40%: 21, 30, 41, 58, 80, 112, 156 and 216. The smaller sets of locators were not subsets of the larger sets of locators. Instead, we placed the locators in each set separately, seeking to distribute the locators strategically to capture the oropharyngeal cavity. We used an alpha value of 4 when performing the endocast method with each locator set. To measure the effect of alpha value on the accuracy of the endocast method, we performed the endocast method using 10 alpha values ranging from 1.8 to 10. Additionally, we allowed the native MATLAB function ‘alphaShape’ to determine a default alpha value. We used the set of 80 locators when performing the endocast method with each alpha value. Lastly, we doubled the alpha shape volumes to get the volume of the full oropharyngeal cavity, and we refer to these values as the ‘endocast volumes’.

Next, as our gold standard method, we measured the volume of air in the oropharyngeal cavity directly from the CT scans. For each CT scan, we first cut the CT scan in half mid-sagittally to mimic the unilateral endocast model. Then, we created a mesh model of the surface of the left half of the head (Fig. 2C). Next, we sealed all openings of the oropharyngeal cavity to create another mesh model that contained all of the tissues of the left half of the head and all of the air inside the oropharyngeal cavity (Fig. 2D). We subtracted the volumes of these mesh models to get the air volume contained in the left side of the head (as visualized in Fig. S2C). Lastly, we doubled the value to get the volume of the full oropharyngeal cavity in each CT scan. We refer to these measurements as the ‘CT air volumes’.

**Fig. 3.**
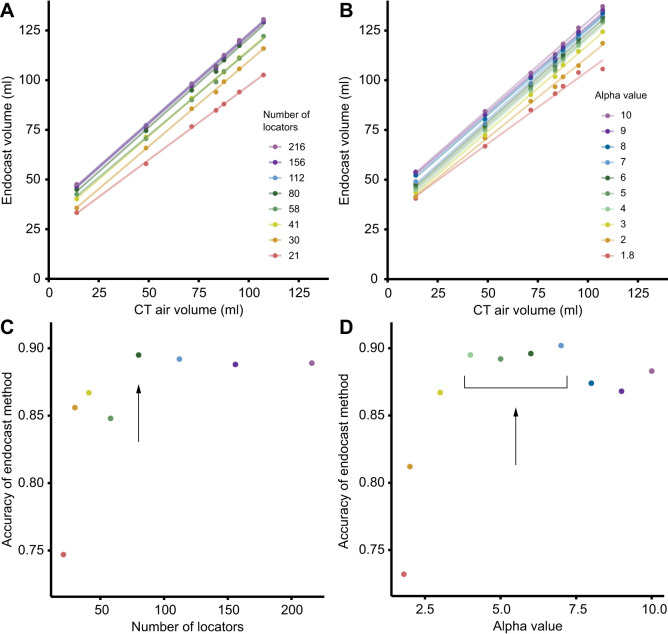
**Effects of number of endocast locators and alpha value on the accuracy of the endocast method.** (A,B) Endocast volume is plotted against CT air volume with each line representing (A) a different number of locators and (B) a different alpha value. (C,D) The accuracy of volume change of the endocast method (slope of each line) is plotted against (C) the number of locators and (D) alpha value. The arrow indicates the ideal number of locators, 80, and alpha values, 4–7, for this study.

To measure the precision and accuracy of the endocast method, we calculated the linear least squares regression of the endocast volume against the CT air volume (Fig. 2E). The *R*^2^ value of the regression line represents the precision of the endocast method, where an *R*^2^ value of one represents perfect precision. The slope of the regression line represents the accuracy of the endocast method at measuring Δ*V*. A slope of one indicates perfect accuracy, a slope less than one indicates that the endocast method is underestimating Δ*V*, and a slope greater than one indicates that the endocast method is overestimating Δ*V*. The *y*-intercept of the regression line measures the volume of soft tissues and bone that is included in the absolute volume of the endocasts. Positive *y*-residual values (i.e. difference in *y*-values between the regression and *y*=*x* lines) indicate that the endocast method is biased towards overestimating the absolute volume as compared with the gold standard method. We calculated the linear least squares regression for each output of the endocast method, with varying numbers of endocast locators and alpha values (Fig. 3).

To better understand how the endocast method performed relative to the CT air volume method, we visualized the endocast meshes and gold standard meshes together in Maya and also used GeoMagic Studio (2014; 3D Systems, Morrisville, NC, USA) to visualize where these volumes were or were not overlapping with each other. For a single CT scan, we used GeoMagic to visualize the volume of air that was in the oropharyngeal cavity but was not included in the endocast (Fig. S2E).

Detailed methods for each of these five steps are provided in Supplementary Materials and Methods.

## RESULTS AND DISCUSSION

Our findings support our hypothesis that the endocast method measures volume change with high precision (*R*^2^=0.998) and with an accuracy of 89.5% (Fig. 2E). The endocast method showed a bias toward underestimating volume change in our simulated suction feeding case study and, as expected, consistently overestimated absolute volume. This overestimation of absolute volume decreased with increasing head expansion because of the underestimation of volume change. Increasing both the number of locators and alpha value improved the accuracy of the endocast method up to a certain point, after which there were diminishing returns or decreases in accuracy, respectively (Fig. 3C,D). While the ideal number of locators and alpha value is likely different for each study, we believe our conclusion that the dynamic endocast method underestimates volume change is likely generalizable to other studies measuring cavities containing a constant volume of hard and soft tissues.

### Absolute volume and change of volume measurement

The endocast method measured absolute volume with high precision (*R*^2^=0.998), but low accuracy (*y*-intercept=31.4 ml, 95% confidence interval, CI [28.2, 34.7]; Fig. 2E). The volume of tissues enclosed in the endocasts was 14.7±0.5 ml (mean±s.d.) for the left half of the head and 29.4 ml for the full endocast volume (Fig. S2F), which is similar to the *y*-intercept of the regression (31.4 ml). These results indicate that the absolute volume of the endocasts consistently included 31.4 ml of tissue. Because soft and hard tissue is functionally incompressible, its constant volume is not expected to affect the accuracy of change in volume measurements. In contrast, the absolute volume of the endocasts is affected by the underestimation of volume change. The inaccuracy of the absolute volume is represented by the *y*-residuals between the regression line and the line *y*=*x* [modeled by *y*=(1−0.895)*x*+31.4]. Thus, the overestimation of absolute volume ranged from 29.9 to 20.1 ml (for the smallest and largest endocasts, respectively).

The volume change results indicate that the dynamic endocast method produced measurements 10.5% smaller than the gold standard method (slope=0.895, 95% CI [0.854, 0.937]; Fig. 2E). This underestimation is attributed to three main regions of oropharyngeal volume omitted by the endocasts: the space under the branchiostegal rays, the space between the ‘lips’ (i.e. the soft tissue covering the premaxilla, maxilla and dentary) and the space along the midsagittal plane (Fig. S2E). We measured the volume of these regions in a single CT scan and found their exclusion from the endocasts contributed to 78%, 10% and 6% of the error in volume, respectively. Visual inspection of the other CT scans suggests a consistent trend. As the oropharyngeal cavity expanded, the volume of those regions increased proportionally, causing an underestimate of change in volume.

We also calculated oropharyngeal volume using a previous endocast method of a deforming polygon (Camp et al., 2015, 2018) and found that it underestimated the change in volume relative to the gold standard (Fig. S3).

### Number of locators and alpha value

Increasing the number of endocast locators increased fit of the alpha shapes to the medial bone surfaces (visual inspection), accuracy of the volume change measurements and accuracy of the absolute volume measurements (Fig. 3A,C). However, more than 80 locators minimally improved accuracy while increasing computational time, making it beneficial to use the lowest number of locators that best capture the volume of interest. Note that while too few locators will generate a smaller *y*-intercept implying a lower overestimate of absolute volume, this is actually caused by smaller than intended endocast volumes that poorly match the surface topology.

Accuracy of the change in volume measurement increased as alpha values increased from 1.8 to 4, plateaued between 4 and 7, and declined with values greater than 7 (Fig. 3A,C). Initially, as alpha values increased, alpha shapes became less concave and better matched the internal cavity. However, alpha values that were too large created alpha shapes that penetrated the bone meshes, reducing accuracy. When we allowed the native MATLAB function ‘alphaShape’ to determine a default alpha value, it selected values between 1 and 2.7, producing alpha shapes that poorly fitted the cranial bones.

### Interpretation of results

#### Study limitations

In contrast to *in vivo* dynamic endocasts, our study is limited by our inability to filter the endocast volume time series and the non-biological abduction of the branchiostegal rays. In typical XROMM studies, enough data points (video frames) are recorded to filter the endocast volume data, reducing noise in the rate of volume change. We only captured seven CT poses simulating cranial expansion, which we believe reduced the precision of our change in volume measurements.

The inaccuracy of change in volume measurements was mostly due to the space under the branchiostegal rays, but this largely reflects an artifact of our specimen manipulation. During *in vivo* suction expansion, the branchiostegal rays are initially passively pulled medially to seal against the pectoral girdle (Li et al., 2022), and only abduct during later stages. Therefore, the accuracy of the change in volume measurements would have been higher if (1) our specimen manipulation more closely matched *in vivo* skeletal motion or (2) the branchiostegal rays had been animated and used to calculate the boundaries of the endocast.

#### Generalizability of results to other endocast studies

The optimal number of locators and alpha value, and precision and accuracy of absolute volume and change in volume, likely differ across species and behaviors. However, we expect the asymptotic relationship between volume change accuracy and increases in the number of locators and alpha value is consistent.

We expect the dynamic endocast method will generally underestimate change in volume because (1) tissues that extend the boundaries of the actual cavity but are not captured by the endocasts will cause the endocasts to underestimate volume change and (2) tissues within the endocasts will not affect volume change if their volume remains constant. Overestimating volume change will likely only happen if the boundaries of the endocast expand (relative to the bones) or if the volume of tissues within the endocast decreases during the behavior. However, because the locators' positions on the bones and the alpha value are fixed, the endocast boundaries should remain consistent. This expectation particularly applies where (1) animated bones and intervening muscles nearly surround the region of interest, (2) the volume of tissues within the endocast is unlikely to change during the behavior and (3) the endocast fit is confirmed by visualization with the XROMM animations.

The overestimation of absolute volume in this study is not generalizable. Absolute volume is determined both by the volume of tissues enclosed within the endocasts (which will bias the endocast volume towards being an overestimate) and the accuracy of volume change (which, if it is an underestimate, will bias the endocast volume towards being an underestimate). If there is little or no soft tissue within the endocast, we expect absolute volume measurements to be more accurate, but to be underestimates if volume change is being underestimated.

The accuracy of absolute volume measurements can be improved by calculating the volume of tissues within the endocast, which our data show is constant, and subtracting this from the endocast volumes of the behavioral dataset (Drost and Van den Boogaart, 1986; Michel et al., 2015; Van Wassenbergh et al., 2009). From a CT scan of a single pose, the endocast and air volume could be calculated and subtracted to obtain the enclosed tissue volume. This would remove the inaccuracy due to enclosed tissues, but volume values would still be affected by inaccuracy in volume change measurements.

### Best practices for measuring volumes with dynamic endocasts

Firstly, determine whether the accuracy and precision of the endocast method is sufficient for your study. Ideally, measure precision and accuracy with this protocol. At minimum, assess whether the assumptions about the relationship between the endocast and the surrounding and enclosed tissues (see previous section) are met.

Secondly, confirm the dynamic endocast method properly captures the volume by viewing the alpha shapes with the XROMM skeletal animations.

Thirdly, determine the optimum number and placement of locators. We suggest iteratively adding locators and generating alpha shapes until the volume of a single endocast plateaus. To determine where additional locators are needed, visually inspect the endocast with the animated bones. For example, locators may be needed in spaces between bones to better define dynamic soft tissue boundaries (see Supplementary Materials and Methods and the tutorial for the dynamic endocast method).

Fourthly, determine the optimum alpha value to capture the bone surfaces with minimal interpenetration. Interpenetration can also be reduced by adding locators. In our study, an alpha value between 4 and 7 worked well; thus we suggest these values as a starting point.

Lastly, recording with a sufficiently high frame rate (capturing the behavior in 50–100 frames) to filter the absolute volume data will reduce noise in its derivative: change in volume data.

### Conclusion

Using a fish oropharyngeal cavity as a case study, we have demonstrated that the dynamic endocast method measures volume change with high precision and accuracy, and that these volume change measurements are conservative (i.e. underestimates). Although the volume of interest in this case study was defined entirely by locators placed on bones, the dynamic endocast method has the potential to be applied more broadly to 3D volumes whose boundaries are defined by both animated bones and soft tissue markers or even by soft tissue markers alone (Hatala et al., 2023). This method also has the potential to generate other measurements, such as quantifying shape change (Olson et al., 2021). While flexibility and broad applicability are strengths of this method, the appropriateness and validity of these applications of the endocast method should be carefully examined.

## Supplementary Material

10.1242/jexbio.249420_sup1 Supplementary information
