# Supplementary material for: Precision and accuracy of the dynamic endocast method for measuring volume changes in XROMM studies
Source: J Exp Biol. 2025 Feb 19;228(4):JEB249420. doi: 10.1242/jeb.249420 (PMC11883290; doi:10.1242/jeb.249420)
Supplement: Supplementary information [file jexbio-228-249420-s1.pdf]

Supplementary Materials and Methods

SUPPLEMENTARY METHODS

Instructions for the dynamic endocast technique are included in the Materials and Methods section of the main paper and a tutorial is provided in the Supplementary Materials. Here we detail the methods used to measure precision and accuracy of the endocast technique relative to CT segmented air volume, our gold standard method. The numbered steps are keyed to those outlined in the Materials and Methods section. In addition, we describe our preliminary analysis of the accuracy of the deforming polygon method, a version of the endocast method that has been used in two prior studies (Camp et al., 2015; Camp et al., 2018).

1. Specimen manipulation and CT scans

The *C. blanci* specimen used here is Cb04, an individual used for *in vivo* XROMM and dynamic endocast measurement of change in volume (delta volume, ΔV) for calculating suction expansion power (Li, Kaczmarek et al., 2022). The body mass of Cb04 was 480 g and standard length was 433 mm.

Postmortem, we supported and immobilized the body of the fish in an upright posture in air, similar to a natural swimming posture in water (Fig. S1). Then, we pulled dorso-caudally on the rostral tip of the neurocranium, which elevated it and expanded the head. We took CT scans at a range of positions between fully closed and fully expanded using a Fidex Veterinary CT Scanner (Animage, Pleasanton, CA, USA) at 0.15 mm isotropic voxel resolution. We selected seven scans that best represented the full range of expansion.

Journal of Experimental Biology • Supplementary information

In these specimen manipulations, we did not attempt to simulate the skeletal kinematics of *in vivo* suction feeding expansion with high fidelity. Rather, we intended to create oropharyngeal expansion that could be used as a sufficient proxy to test the dynamic endocast method. The simulated expansion differed from *in vivo* suction expansion in four main ways: 1) no *active* lateral or ventral expansion, 2) no *active* pectoral girdle retraction, 3) the opercular membranes and branchiostegal rays were allowed to open (Fig. S1), and 4) the maximum magnitude of oropharyngeal expansion was greater. The first two differences were minor because lateral and ventral expansion and pectoral girdle retraction occurred passively: the lower jaw and hyoid pushed against the suspensorium, causing lateral expansion of the suspensorium and operculum, and the extreme amount of neurocranial elevation caused the lower jaw, hyoid bars, and pectoral girdle to retract relative to the neurocranium (rather than relative to the body). The third difference stems from the observation that, during *in vivo* suction expansion, the opercular membranes and branchiostegal rays seal against the pectoral girdle (likely passively), reducing the volume of oropharyngeal expansion (Li et al., 2022). This difference was partially addressed because the opercular membrane was not captured by the dynamic endocast method and was excluded from the gold standard method. Lastly, the simulated maximum volume, as measured by dynamic endocast technique (120mL), was 20% larger than the largest *in vivo* volume (100mL).

2. Dynamic endocast measurements of oropharyngeal volume

We measured the oropharyngeal volumes in the CT scans using the dynamic endocast method for comparison with the CT air volumes. Cb04 had at least three radio-opaque bone markers implanted in the neurocranium, maxilla, lower jaw, suspensorium, operculum, ceratohyal, and cleithrum (all paired bones were marked on the left side of the head). We used the XROMM Maya Tool (XMT) ‘vertAvg2’ (https://bitbucket.org/xromm/xromm_mayatools/) to measure the XYZ coordinates of the radio-opaque markers in the seven CT scans in Autodesk Maya (2018; Autodesk, San Rafael, CA, USA). Then, we concatenated the seven sets of XYZ coordinates into a pseudo time series simulating seven time steps and calculated rigid body transformations. We made mesh bone models from one of the CT scans and then animated the bone models to create a pseudo-dynamic XROMM animation of the seven expansion poses.

We used the dynamic endocast technique to measure oropharyngeal volume in the pseudo-dynamic XROMM animation using the methods described generically in the Materials and Methods section of the main paper and with more specificity in the *in vivo* study (Li et al., 2022).

Locators were placed and parented creatively in order to effectively and reliably define the boundaries of the oropharyngeal cavity (Fig. 2A). The locators on the bones were distributed approximately evenly and placed strategically to capture the shape of the space. Locators placed on the caudal edge of the operculum and the rostral edge of the cleithrum defined the back of the oropharyngeal cavity. In addition to placing locators directly on the oropharyngeal surfaces of the animated bones, locators were placed along the midsagittal plane, a plane created in Maya (native Maya function) that was aligned to the midsagittal plane of the head as defined by the orientation of the neurocranium. Locators were also placed in between bones where necessary (between the ventral tips of the lower jaw, ceratohyal, and cleithrum,

mimicking the borders formed by the sternohyoideus and protractor hyoideus muscles (Fig. 2A)). These locators were necessary to prevent the alpha shapes from occasionally forming gaps in those regions due to low locator density when the head was expanded. In order to animate these locators realistically, the locators were parented to cylinders that had one end constrained to the ventral tip of one bone, while the other end always dynamically pointed to the ventral tip of the adjacent bone (see the tutorial for greater detail).

Then, 3D coordinates of the locators were exported from Maya and imported into MATLAB (R2020a; The Mathworks, Natick, MA, USA). A custom-written script (available at https://bitbucket.org/ArielCamp/dynamicendocast) read in the 3D coordinates of the locators and generated an alpha shape for each CT pose. An alpha shape is a method of triangulation that uses a specified radius, or an alpha value that represents the inverse of the radius, to generate a bounding shape with control over how tight the bounding shape wraps around the point cloud, i.e. the coordinates of the locators (Edelsbrunner and Mücke, 1994). Once alpha shapes have been generated for each time step, the script also calculates their volumes. The alpha shapes were exported as polygonal mesh models and imported into Maya for visualization (Fig. 2B). The volumes of the alpha shapes (left side of the head) were doubled to obtain the bilateral oropharyngeal volume, which we will refer to as the 'endocast volume.'

We repeated the endocast method multiple times while independently varying the number of endocast locators placed and the alpha value used in order to investigate their effects on the accuracy of the endocast method. We used eight sets of locators, where the number of locators in each set varied from 21 to 216, increasing by 40%: 21, 30, 41, 58, 80, 112, 156, and 216. The smaller sets of locators were not subsets of the larger sets of locators. Instead, we placed the locators in each set separately, seeking to distribute the locators strategically to capture the oropharyngeal cavity. We used an alpha value of 4 when performing the endocast method with each locator set. To measure the effect of alpha value on the accuracy of the endocast method, we performed the endocast method using 10 alpha values ranging from from 1.8 to 10. Additionally, we allowed the native MATLAB function 'alphaShape' to determine a default alpha value. We used the set of 80 locators when performing the endocast method with each alpha value.

3. Gold standard measurements of oropharyngeal volume

We measured the volume of air in the oropharyngeal cavities in the CT scans as gold standards to compare to the endocast volumes. We did this in four steps: 1) measuring the volume of the left side of the fish's head, 2) measuring the volume of the left side of the fish's head as well as the air inside the oropharyngeal cavity, 3) subtracting these volumes to obtain the volume of air inside the left side of the oropharyngeal cavity, and 4) doubling the resultant volume to measure bilateral oropharyngeal volume, as was done using for the dynamic endocast method.

We produced 3D polygonal mesh models from CT scans with the open-source medical imaging software Horos (Purview, Annapolis, MD, USA). We used a -500 threshold to include all hard and soft tissues in our models. We then imported these mesh models into GeoMagic Studio (2014; 3D Systems, Morrisville, NC, USA).

Journal of Experimental Biology • Supplementary information

For the gold standard method of measuring volume to be most comparable to the endocast method, both needed to be defined by the same oropharyngeal cavity and midsagittal plane boundaries. Therefore, we exported the cleithrum, operculum, and midsagittal plane mesh models from the endocast Maya scene, retaining their animated positions for each CT scan, and imported them into GeoMagic with each of the thresholded mesh models. Each mesh model was trimmed at its caudal end, using the imported cleithrum and operculum models to visualize and define the same boundaries that were used in the endocast method. This excluded the flexible opercular membrane that extends from the operculum, however this membrane moves passively during suction feeding and does not contribute to oropharyngeal expansion (E.B.K. personal observation). Then, the mesh model was divided into left and right halves along the midsagittal polygon plane (imported from the endocast method) using the 'trim with plane' tool, the right half was removed. This produced a closed mesh model that surrounded the soft and hard tissues of the fish head, which we will refer to as a 'surface mesh' (Fig. 2C).

Each surface mesh was then duplicated so that we could make a second model that surrounded the air inside the mouth, as well as the hard and soft tissues (Fig. 2D). In the second model, we bridged and filled all openings into the oropharyngeal cavity to effectively 'shrink wrap' the entire fish head (including the gaps between the mandible, maxilla, and premaxilla, the operculum and cleithrum, and the dorsal and ventral perimeters of the oral cavity along the midsagittal plane (producing a complete midline wall)). We then deleted all internal mesh components to produce a shrink wrap shell of the left side of the head (Fig. 2D). We refer to this as a 'wrapped mesh'.

We calculated the volume of each model using the volume tool in GeoMagic. We then subtracted the volume of the surface mesh from the volume of the wrapped mesh to calculate the volume of air within the left half of the oropharyngeal cavity in each of the seven CT scans (as visualized in Fig. S2C). We then doubled this volume to obtain the volume of the full oropharyngeal cavity, which we will refer to as the 'CT air volume.'

4. Precision and accuracy of the endocast method relative to the gold standard method

The dynamic endocast method is designed to measure the *change in volume* (ΔV) over time, not the *absolute volume* at any given time step, which is expected to be overestimated by the endocast method. To measure the precision and accuracy of the endocast method, we sought to quantify how well it captures *change in volume* relative to the gold standard method.

We calculated the linear least squares regression of the endocast volumes against the CT air volumes (Fig. 2E, Fig. 3A,B). As described in the Materials and Methods section of the main text, the R^2 value of the regression line represents the precision of the endocast method, where an R^2 value of one represents perfect precision. The slope of the regression line represents the accuracy of the endocast method at measuring ΔV. A slope of one indicates perfect accuracy, a slope less than one indicates that the endocast method is underestimating ΔV, and a slope greater than one indicates that the endocast method is overestimating ΔV. The y-intercept of the regression line measures the volume of soft tissues and bone that are included in the absolute volumes of the endocasts. Positive y-residual values (i.e., difference in y-values between the

Journal of Experimental Biology • Supplementary information

regression and y=x lines) indicates that the endocast method is biased towards overestimating the absolute volumes as compared to the gold standard method. The endocast method is expected to overestimate the absolute volumes because the endocasts are generated from locators on the surfaces of the bones, and so the endocast includes the volume of hard and soft tissues that are internal to those bone surfaces.

To examine how the number of endocast locators and the alpha value influence the accuracy of the endocast method, we regressed the endocast volumes against the CT air volumes for each iteration of the endocast method, for which we varied in the number of locators and the alpha value (Fig. 3A,B). We plotted the slope of those regression lines against either the number of locators placed or the alpha value used (Fig. 3C,D).

5. Visualization of endocast method relative to gold standard method

To better understand how the endocast method performed relative to the gold standard method, we visualized the alpha shapes and gold standard meshes together and created new mesh models to visualize their intersections. This allowed us to confirm that the midsagittal plane and caudal boundaries were defined similarly in both methods and see where the wrapped mesh was capturing more volume than the endocast meshes.

Then, using the CT scan of the oropharyngeal cavity with the greatest expansion, we used the boolean tool in GeoMagic to create a mesh of the air inside the head by subtracting the surface mesh from the wrapped mesh (Fig. S2C). Next, we subtracted the alpha shape from the newly created air mesh to visualize regions of air outside the endocast (Fig. S2E).

Additionally, for each CT scan, we used the boolean tool in GeoMagic to visualize the soft and hard tissues that were inside the bounds of the endocasts by creating meshes from the intersection of the alpha shapes and the surface meshes (Fig. S2F).

Preliminary analysis of the deforming polygon endocast method

For comparison to previous studies (Camp et al., 2015; Camp et al., 2018), we also calculated the volume using a deforming polygon endocast. In this method, a single polygon is created and its vertices are linked to locators on the bones so that the polygon 1) fits the mouth cavity and 2) deforms as the bones surrounding the mouth cavity move. The volume of this single polygon is then calculated at each frame of the animation (Camp et al., 2015; Camp et al., 2018). For this dataset, a sphere with 92 vertices was created, and each vertex was linked (point constrained) to at least one bone locator and/or other vertex at a single pose of the knifefish specimen. The volume of the deforming polygon was calculated at each of the seven expansion poses using a custom MEL script (written by SMG). Both the visualization and volume calculation of the deforming polygon endocast were done in Maya.

Like the alpha shape endocast, the deforming polygon endocast overestimated the absolute volume and underestimated the change in volume relative to the gold standard (Fig. S3).

Journal of Experimental Biology • Supplementary information

However, with regards to absolute volume, the deforming polygon endocast had a lower y-intercept and, therefore, did not include as large of a volume of soft tissues and bone as did the alpha shape endocast. This likely indicates that the deforming polygon endocast was not as tightly fit to the internal surfaces of the bones. (Endocasts have positive y-intercepts (of the regression of endocast volumes to the gold standard volumes) because they surround soft and hard tissue in addition to oropharyngeal volume. Endocasts that are poorly fit to the inside of the bones have lower y-intercepts. See, for example, the regression lines of endocasts with few locators or low alpha values, Fig. 2.) This is not surprising because the deforming polygon endocast was not as refined as the alpha shape endocast.

For this knifefish dataset, the deforming polygon endocast was less accurate at measuring the rate of volume change than the alpha shape endocast. The slope of the linear least squares regression line of the endocast volumes as a function of CT air volumes was 0.81 for the deforming polygon endocast indicating that it underestimated the rate of volume change by 19%. For comparison, the alpha shape slope was 0.9, indicating an 11% underestimation. We are uncertain if the deforming polygon method is consistently less accurate across different applications of the endocast method. But this suggests both endocast methods give broadly similar results, lending confidence to previous studies (Camp et al., 2015; Camp et al., 2018).

This analysis used a relatively simple application of the deforming polygon method, and more tailored, customized polygons likely provide better fits. Specifically, in this preliminary analysis and in prior studies (Camp et al., 2015; Camp et al., 2018), the initial shape of the deforming polygon was a sphere (before its vertices were constrained and manipulated). The nature of a spherical polygon may make it more difficult to mold the sphere to the region of interest, and a custom-built polygon, in which the vertices of each face are designated individually, may perform better (Hatala et al., 2023). While it is possible that alpha shapes adapt to shape changes better than the fixed faces of deforming polygons, deforming polygons offer a consistent set of faces and direct control of fit, which may be advantages for many research questions.

Journal of Experimental Biology • Supplementary information

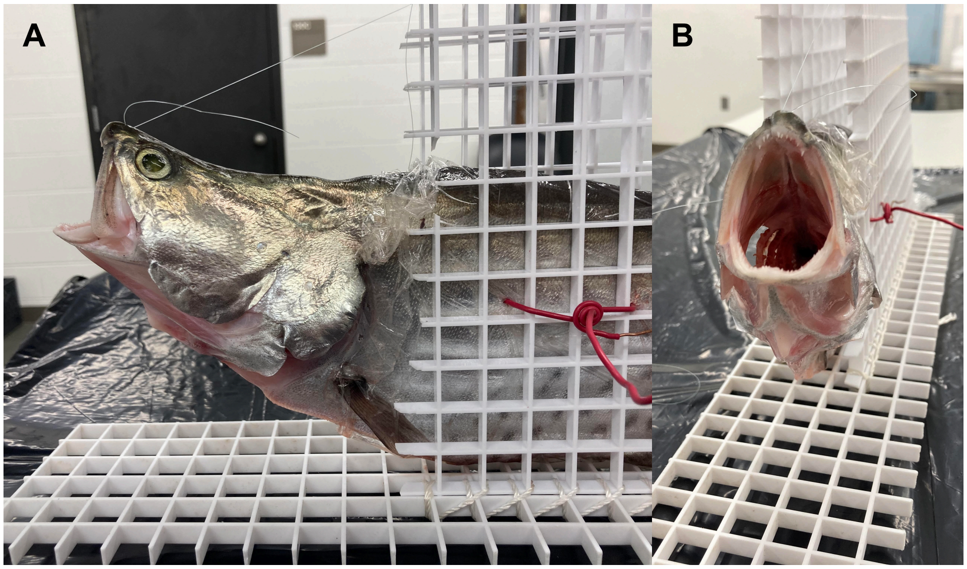

Fig. S1. Specimen manipulation. (A) Lateral and (B) anterolateral views of the euthanized *Chitala blanci* specimen during CT scanning. The specimen was supported and immobilized in an upright posture while its neurocranium was elevated, causing lateral and dorsoventral expansion of the head. The amount of neurocranial elevation was varied between scans.

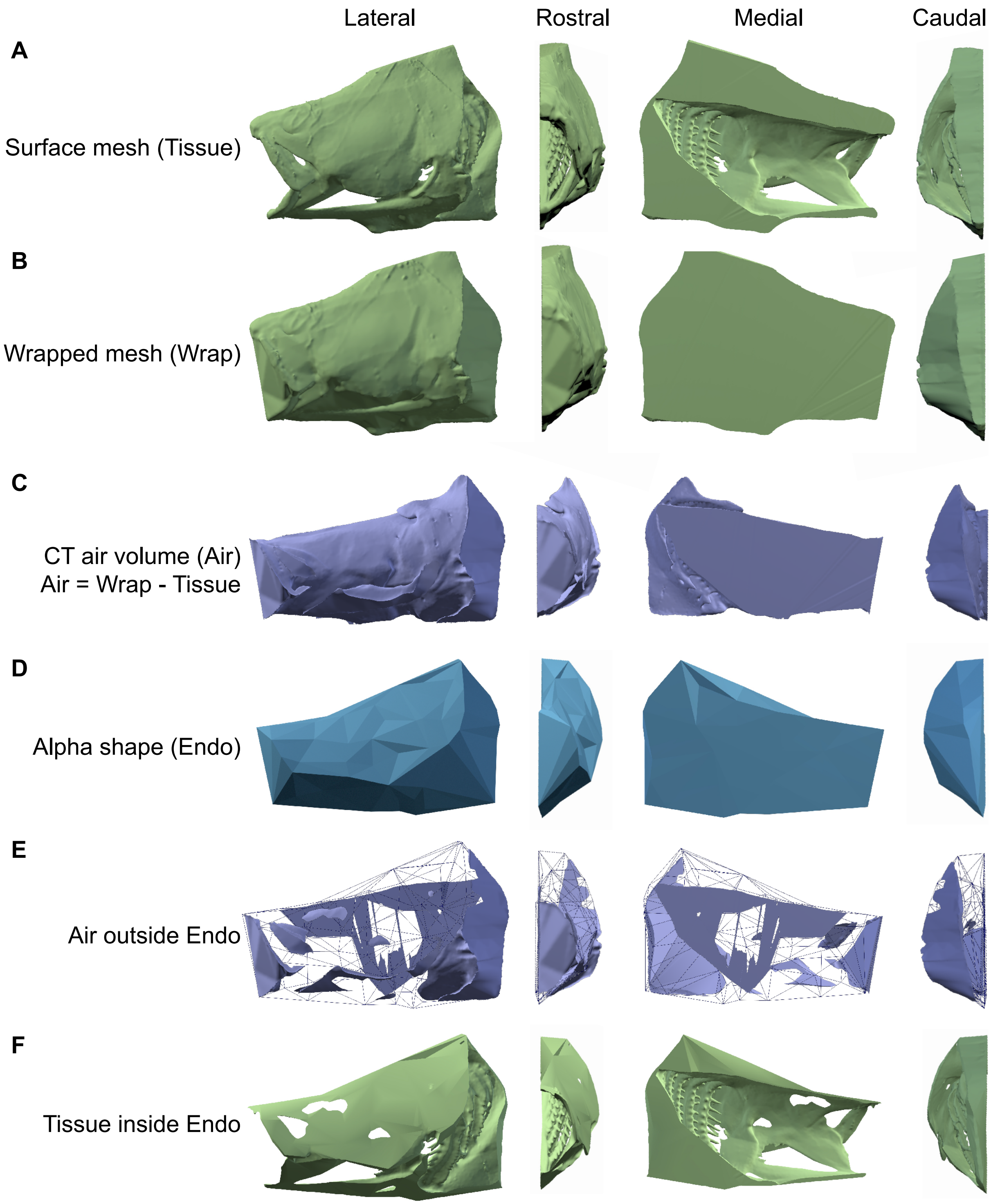

Fig. S2. Dynamic endocast and CT air volume meshes. The CT air volumes were calculated by subtracting the volume of the surface mesh from the volume of the wrapped mesh. This numeric subtraction is visualized here as a subtraction of the polygonal meshes from one of the CT scans. The surface mesh (A) subtracted from the wrapped mesh (B) produces a mesh of the air inside the buccal cavity (C). The air inside the buccal cavity (C) that was outside of the alpha shape (D) is visualized (E), with a wireframe of the alpha shape. The soft and hard tissues of the head (A) that were inside the alpha shape (D) are visualized as well (F).

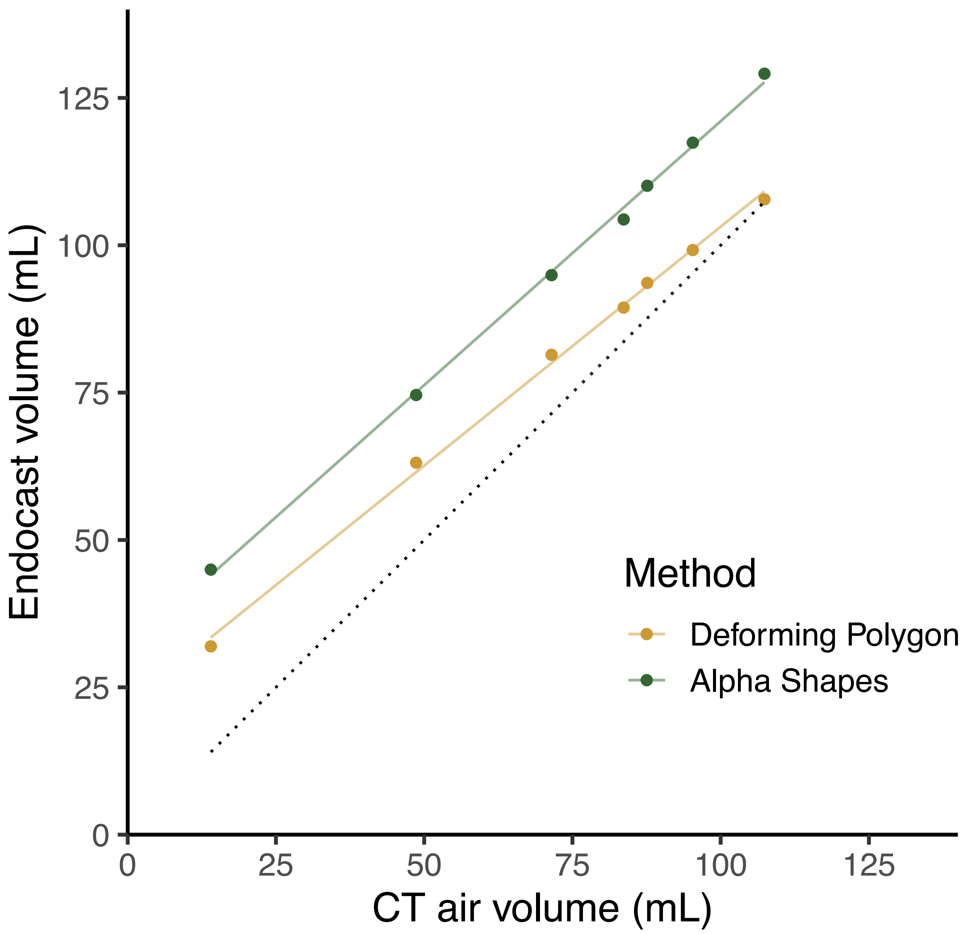

Fig. S3. Comparison of endocast methods. The endocast volume measured with the deforming polygon method (yellow points) are plotted as a function of CT air volumes. The solid line illustrates the line of best fit and the dotted line illustrates a 1:1 relationship. The alpha shape endocast volumes and best fit line from Fig. 2E are also included for comparison.

Journal of Experimental Biology • Supplementary information

Tutorial for the Dynamic Endocast Method

Overview of the dynamic endocast method

The ‘dynamic endocast’ method is used to measure the change in volume of a 3D space bounded by bones in an XROMM animation. This is useful for studying how a 3D region, through which fluid (air or water) is moving into and/or out of, changes volume during a behavior. Dynamic endocasts are created by placing virtual landmarks (i.e., locators) on the medial surfaces of animated bone mesh models. The locators move with the bones, and their dynamic 3D coordinates are used to define the shape of the endocast at every frame of the animation. An alpha shape, a type of convex hull, is used to create a mesh that wraps around the locators to create a shape that encloses the 3D space defined by the medial surfaces of the bones.

In using this method, we acknowledge that in addition to capturing the volume of interest, the alpha shapes likely include the surrounding soft tissues and/or unanimated bones. However, if the volume of soft and hard tissues inside the alpha shapes remains constant throughout the animation, they will not affect measurements of volume change. These alpha shapes are generated frame-by-frame from the XROMM animation, thus producing a time-varying, dynamic endocast, from which instantaneous rate of volume change can be determined.

The assumptions and limitations, as well as accuracy and precision, of the endocast method are discussed by Kaczmarek et al. (2025). We highly recommend reading that paper before deciding whether it is appropriate to apply the dynamic endocast method to address your research question. The dynamic endocast method was designed for measuring instantaneous volume change (ΔV/dt). Although other measurements may be obtained using this method (e.g. absolute volume or 3D shape changes), these other measurements may have consistent biases (as demonstrated for absolute volume by Kaczmarek et al. (2025)) and must be used with caution. Absolute volume measurements obtained using this method can be improved by measuring the volume of tissue enclosed in an endocast created from a CT scan of a single pose and subtracting this volume from the *in vivo* endocast volumes. Before applying the dynamic endocast method, and again before interpreting and disseminating their results, we recommend consider whether 1) the animated bones and intervening muscles come close to fully bounding the region of interest, and 2) the volume of soft tissues and bones within the endocast remains constant during the animations.

Preparation for this tutorial

1. Install Autodesk Maya, MATLAB, and XROMM Maya Tools (XMT).
   - Autodesk Maya can be downloaded here (free for students and educators):
     https://www.autodesk.com/education/edu-software/
   - MATLAB can be downloaded here (paid license required):
     https://www.mathworks.com/products/matlab.html
   - XROMM_MayaTools can be downloaded here:
     https://bitbucket.org/xromm/xromm_mayatools/src/master/

   - The XROMM_MayaTools wiki page can be found here:

Journal of Experimental Biology • Supplementary information

https://bitbucket.org/xromm/xromm_mayatools/wiki/Home

2. Download the tutorial files: https://bitbucket.org/ArielCamp/dynamicendocast
    - Endocast_Tutorial_Ref_Scene.ma (Maya reference scene, containing example endocast locators)
    - Endocast_Tutorial_RBT.csv (rigid body transformations to create animated scene)
    - expTransWSbyFrame.mel (Maya script for exporting XYZ coordinates from Maya scene)
    - dynamicEndocastBeta.m (Matlab script for calculating alpha shape objects and volumes)
    - impHulls.mel (Maya script for importing alpha shape objects into Maya scene)
    - Example files:
        - Endocast_Tutorial_Example_Animation_Scene.ma (Maya animation with alpha shapes created from example endocast locators)
        - Endocast_Tutorial_Example_Locator_Coordinates.csv (3D coordinates of example endocast locators)
        - Endocast_Tutorial_Example_Alpha_Shapes.zip (alpha shapes exported from MATLAB, created from example endocast locators)
        - Endocast_Tutorial_Example_Volume_Data.csv (volume data exported from MATLAB, created frome example endocast locators)

3. Load Maya scripts
    - **Windows:** move the two .mel scripts into the following directory
    - C:\Documents and Settings\User_Account\My Documents\maya\scripts
    - **Mac OS:** move the two .mel scripts into the following directory (you will have to hold down the Option key and click on the Go menu in the Finder to see the Library folder)
        - MacintoshHD/Users/User_Account/Library/Preferences/Autodesk/maya/maya Version(e.g. 2023)/scripts
        - It also works to place them in the following directory: MacintoshHD/Users/ User_Account/Library/Preferences/Autodesk/maya/scripts
    - Restart Maya, and type ‘rehash’ into the MEL command line

4. Load Matlab script
    - Move the .m script into whatever folder you use to keep your Matlab scripts, OR set a new path in Matlab to the folder where the .m script is currently.

5. Review the glossary at the end of this document

Overview of this tutorial

This tutorial explains how to create a dynamic endocast in an XROMM animation, using the XROMM knifefish dataset from Li et al. (2022), which measured oropharyngeal cavity volume change to study suction feeding power. We have broken this process into the following steps:

1. Create reference scene in Maya
2. Place endocast locators in reference scene
3. Animate the bones in the animated scene
4. Export coordinates of the endocast locators from Maya

Journal of Experimental Biology • Supplementary information

5. Calculate alpha shapes in MATLAB
6. Import alpha shapes into Maya
7. Improve the fit of your alpha shapes

Within this tutorial, we highlight terms that refer to actions that can be taken in Maya (e.g. parenting), and we provide a glossary of the highlighted terms at the end of this document with explanations of how to perform those actions and what they do. When there are multiple ways to accomplish a task, we list those options within a box. “Tip:” indicates that a task is optional, not essential, and “**IMPORTANT:**” emphasizes critical steps.

Throughout the tutorial, we will switch between a Maya scene that contains the bones and endocast locators (‘reference scene’) and a separate Maya scene in which those bones are animated using the rigid body transformations from a single trial (‘animated scene’). The animated scene does not contain the bones and locators themselves – instead it ‘references’ the reference scene. The data in the reference scene is ‘read in’ each time that the animated scene is opened.

Although it is not necessary to set up Maya scenes this way, it is beneficial because endocast locators only have to be placed once for each animal studied, since multiple animation scenes (i.e. for all of the trials recorded from that animal) can utilize the same reference scene. Another benefit is that any changes made to the reference scene are automatically propagated onto the animation scenes that reference it. A limitation is that you cannot see how the objects in the reference will move in an animation without closing it and reopening the animation scene. Therefore, it is important to open up an animation scene after making changes to the reference scene to confirm that objects are moving as expected.

1. Open reference scene in Maya

Open the scene ‘Endocast_Tutorial_Ref_Scene.ma’ in Maya. This scene contains the bone mesh models and a virtual plane aligned to the midsagittal plane of the neurocranium. The knifefish dataset used for this tutorial only has unilaterally animated bones, so we created a virtual midsagittal plane to capture unilateral volume, which will later be doubled to represent the total oropharyngeal volume. The midsagittal plane is initially hidden but it can be revealed by changing the visibility of the layer that contains it (Channel Box/Layer Editor window < Display tab < click on the left-most square next to the layer ‘midsagittal plane’).

Additionally, this scene contains a pre-made set of endocast locators that surround the volume of interest (the oropharyngeal cavity of a knifefish). These locators are in the layer ‘example_endocast_locators’ and can be revealed by clicking the left-most square next to the layer name (see end of Step 2 for more detail). We recommend waiting until you have tried placing your own locators (Step 2) before you look at this pre-made set.

To create a reference scene with your own data, simply import the .obj file (using XMT ‘imp’) for each bone into a new Maya scene. If your dataset has bilaterally animated bones, then the bilateral volume can simply be captured by placing locators (Step 2) on the medial surfaces of

Journal of Experimental Biology • Supplementary information

all of the bones. If your dataset has unilaterally animated bones, then you will first need to define a virtual midsagittal plane by creating a rectangle polygon (Create < Polygon Primitives < Plane), manually moving and aligning it to the midsagittal plane of the bones, and parenting the polygon plane to the appropriate bone (e.g. neurocranium).

2. **Place endocast locators in reference scene**

<u>2a. Create initial endocast locators</u>

To create a locator, you can either click Create > Locator, or you can click on the purple locator symbol between ‘exAnim’ and ‘Hgph’ in the XMT shelf. Additional locators can be created in the same way, or by duplication of an existing locator.

Tip: We recommend adding the first endocast locator to a layer, and then duplicating that locator to create additional locators. New duplicated locators will also be members of that layer.

Tip: We recommend naming the first locators that will be placed on each bone according to the bone’s name (e.g Neurocranium_1) and parenting them to their respective bones. Then, duplicate this first locator to create additional locators for each bone. Maya will automatically name the new locators with a higher numerical suffix (e.g. ‘Neurocranium_2’) and keep the same parenting relationship as the original locator.

Tip: To see where a locator is located, select the locator and then press ‘f’ while hovering your mouse in the Maya ‘viewport’ (i.e. the main window displaying your 3D scene). The camera will center (‘focus’) itself on the position of the locator.

<u>2b. Place endocast locators on the surfaces of the bones</u>

The goal of placing endocast locators is to best cover the surface of the internal cavity without using an unnecessary number of locators. As demonstrated by Kaczmarek et al. (2025), increasing the density of locators only improves the quality of the endocast shape to a point, after which there are marginal returns. Make sure to place locators that capture variation in the concavity of the bone’s medial surface and any protrusive landmarks. The density and placement of these locators will alter the amount of interpenetration of the endocast volume and the bones.

Locators can be translated using the ‘Move’ tool.

Tip: Use the ‘snap to vertex’ tool: select the locator, switch to the ‘Move’ tool, and hold down ‘v’. This allows the locator to move along the vertices of all visible meshes.

Tip: For each bone, we recommend first placing locators around the border of the bone to capture its general geometry and then placing additional locators on the medial surface of the bone, attempting to capture its surface morphology. Avoid increasing the density of the locators around the border, especially along a linear border, as this may only improve the quality of the endocast shape marginally and while creating redundancy, slowing down Maya and Matlab.

Journal of Experimental Biology • Supplementary information

2c. Place endocast locators on the midsagittal plane

The goal is to distribute locators throughout the midsagittal cross-section of the oropharyngeal cavity without using an unnecessary number of locators.

Locators can be translated using the 'Move' tool.

Options. Here are some helpful tools for placing locators on the midsagittal plane:

- Use the 'live object' tool. This only allows the locator to move over the surface of the plane.
- Use the 'snap to vertex' tool. This only allows the locator to move along existing vertices of all existing meshes. Be careful not to accidentally snap the locator to the vertices of the bones instead of the plane.
- Use a point constraint to position a locator exactly in the middle of two other locators. To do this, select two other locators and then select the locator that you want to move. While in the 'Animation' menu bar, click Constraint > Point. **IMPORTANT**: once the locator is in the proper position, delete the constraint (it is a node parented under the locator).
    - If the locator does not move as expected, then open up the settings for the point constraint (Constraint > click on the square to the right of 'Point'). Make sure that 'Maintain offset' is unchecked, that the offset is zero for all axes, and that 'Constrain axes' is set to 'all'

Tip: We recommend first placing locators along the border of the midsagittal cross-section of the oropharyngeal cavity. We then recommend placing locators along the inside of the border as evenly spaced as possible.

IMPORTANT: The locators must be placed such that they are within the midsagittal cross-section during the entire animation, including when the cavity is at its smallest volume (otherwise they will cause the endocast to extend outside of the volume of interest). In this tutorial, the mouth of the knifefish is closed in the CT scan (and reference scene), so locators that are placed within the midsagittal cross-section in the reference scene will stay within the midsagittal cross section throughout the entire animation.

2d. Parent endocast locators to their associated objects

If you have not already done so, parent the locators to the bone that they are placed on (locators along the midsagittal plane should be parented to the neurocranium). Confirm that all of the locators are parented to the correct object.

2e. Put endocast locators into a layer

If the endocast locators are not already all members of the same layer, we recommend adding them to a layer. This will allow you to hide and reveal the locators (clicking the left-most square next to the layer name: 'V' for view and empty for hide) and allow you to select all of the locators easily (right-click on the layer name and click "select objects").

- Select all of the locators that you have placed.

Journal of Experimental Biology • Supplementary information

Options. Here are a few ways to select all of the locators:
- In the main window (with the example locator set hidden), hold shift and drag a box around all of the bones and locators. In the outliner or hypergraph hierarchy, deselect the bones one by one.
- In the outliner window or in the hypergraph hierarchy, select all of the locators that you placed (but not the locators that are part of the example locator set)
- First select one locator and then click Select > Similar. This will select all locators in the scene, even those that are hidden, such as the example endocast locators that are in this tutorial maya scene. So, if you use this option, you would then have to deselect the example locators (in the outliner or hypergraph hierarchy). This option is best used when working with your own dataset, not this tutorial.

- While all locators are selected, add them to a new layer by clicking on the rightmost icon (gray rectangle with a blue circle) in the Layer Editor window.
- Double click the label ‘layer1’ and name it ‘Endocast_Locators.’

To add locators to the layer after the layer has been created, select the locators, right-click on the layer, and select ‘add selected objects.’

2f. Save the reference scene with your endocast locators.

2g. Look at the example locator set

Finally, compare your locator placement to the example locator set provided in the ‘example_endocast_locators’ layer.

3. Animate the bones in the animated scene

After placing the endocast locators on the bones in our reference scene, animate those bones using rigid body transformations, which are often exported from XMALab.

3a. Create an animation scene.

Save and close the reference scene that you placed endocast locators in (‘Endocast_Tutorial_Ref_Scene.ma’). Open a new Maya scene and save it as ‘Endocast_Tutorial_Animation.ma.’ Add ‘Endocast_Tutorial_Ref_Scene.ma’ as a referenced file (File > Create reference > find ‘Endocast_Tutorial_Ref_Scene.ma’ > click Open), and then edit the reference scene namespace (File > Reference editor > click on ‘Endocast_Tutorial_Ref_Scene.ma’ > rename the ‘namespace’ to something short (e.g. ‘r’) and press enter).

3b. Set keyframes.

Tip: We recommend setting keyframes for the bones at frame 0 and at the first frame before the bone animation starts (this needs to be done before animating the bones (Step 3c)). Setting these keyframes keeps the bones in the same positions that they are in in the reference scene, for the frames before the animation. We recommend doing this so that it is easy to view the

Journal of Experimental Biology • Supplementary information

bones in their reference pose without needing to close the animation scene and opening the reference scene.

Select all of the bones. Change the current time to zero and set the keyframe. Change the current time to 349 (in the tutorial dataset, bone animation starts on frame 350) and set the keyframe.

3c. Animate the bones.

To animate the bones, import the rigid body transformations (RBTs) and apply them to their respective bones.

Options. Here are two ways to import the rigid body transformations (RBTs):

- Single file: RBTs can be exported from XMALab as a single file, with three columns for each bone.
    - The benefits of using a single file include that there is only one file for each trial, making these files easier to organize, and that all of the RBTs can be imported in Maya at the same time.
    - A disadvantage of using a single file is that, when you import the RBT file into Maya, you have to carefully select the bones in the order that bones are listed in the file. In addition, if the RBT file includes columns for bones that you do not want to animate in the Maya scene, it can be difficult to subset the correct columns during import.
- Separate files: RBTs can be exported from XMALab as separate files for each bone.
    - The benefit of using separate files is that it is simple to match each bone with its corresponding RBT.
    - The disadvantages of using separate files are that there are more files to keep organized and up to date, and it requires selecting each bone and loading in its respective file when importing the RBTs into Maya.

In this tutorial, we provide a single RBT file ('Endocast_Tutorial_RBT.csv'). To animate the bones using this RBT file, first, select the bones in the following order: neurocranium, suspensorium, ceratohyal, cleithrum, maxilla, operculum, lower jaw (this is the order of their columns in the RBT file). Using the XMT 'imp' function, load the RBT file path, select 'Rigid Body Matrix,' and confirm the following settings: type = 'global,' 'Column Headers in Row: 1' is checked, 'Rows are time series' is checked, 'Transform Type Between Rows' is 'absolute', and 'Apply to: selected object' is selected. Close the window and play through the animation to make sure the bones are moving properly.

Tip: When working with your own dataset, if you import the RBTs in a single file, we recommend changing the order of the bones in the 'Outliner' window in your reference scene to match the order of the bones in the columns of the RBT files. This makes it easy to select the bones in the correct order when importing the RBTs in your animated scenes.

Tip: When working with your own dataset, if you import the RBTs in a single file, and you have bones in the RBT file that are not present in the Maya scene, make sure that you list the correct

Journal of Experimental Biology • Supplementary information

column range of the RBT file in ‘selected subset’ of the import box. However, we note that we have sometimes experienced errors when selecting a subset of columns, and we recommend importing RBTs as single files if you have difficulty with this.

4. **Export coordinates of endocast locators from Maya**

Save your file before exporting, in case Maya freezes during export.
Select all of your endocast locators by right clicking on the endocast locator layer (‘Endocast_Locators’) in the ‘layer editor’ panel and clicking ‘Select all objects.’ Confirm that all of your endocast locators are highlighted, but that none of the bones, other objects, or pre-made example locators are selected. Then export the translation (XYZ world space position coordinates) of the selected endocast locators, either using the ‘expTransWSbyFrame.mel’ script or the XMT ‘exp’ script. Both methods will output a .csv file of locator coordinates as a function of time, with frame number in the first column and locator names in the first row.

Tip: For this tutorial, we recommend exporting frames 400-450 in order to minimize the computation time in Maya and Matlab. When working with your own dataset, export all animated frames in the trial.

Options. Here are two ways to export the endocast locator coordinates:

- ‘expTransWSbyFrame.mel’ script: Type ‘expTransWSbyFrame’ in the MEL command line and press ‘enter’ (if this results in an error, check that the ‘expTransWSbyFrame.mel’ file is in the correct location on your computer, following the instructions at the beginning of this tutorial). Enter the frame range that you want to export, and then name and save this .csv file.
    - Using ‘expTransWSbyFrame.mel’ will provide a faster export from Maya.
    - **IMPORTANT:** This script will output the position of each locator even for the unkeyed frames (i.e. if there are unanimated frames within your specified frame range, these rows will contain the position of the locators in world space, not NaNs). It is highly recommended that you are careful to only export frames in which the bones are animated.

- XMT ‘exp’ script: Click on ‘exp’ in the XMT shelf, select ‘Translation,’ check ‘Column Headers,’ ‘Row Headers (frame#),’ and ‘Keep frame spacing with NaNs,’ select ‘single file,’ check ‘Advanced Options’ and select ‘Hierarchy of selected objects’, and then specify the frame range you want to export (note: specifying the frame range is the last step).
    - **IMPORTANT:** This script will output the position of each locator as ‘NaN’ during unkeyed frames, and will include all NaN rows for all frames prior to the first frame of the specified frame range. Before reading this file into MATLAB, you must open the .csv output file and delete the rows prior to the animation, otherwise the ‘dynamicEndocastBeta.m’ script will produce an error.

IMPORTANT: If you use constraints to dynamically position your locators as described in Step 7b (i.e. the locators aren’t simply parented to an animated bone and instead are parented to an object that must remain constrained in order to be properly positioned), then you MUST use ‘expTransWSbyFrame’ instead of ‘exp.’ The file output by the XMT ‘exp’ function with ‘Hierarchy of selected objects’ selected will have ‘NaN’ values for all frames of

Journal of Experimental Biology • Supplementary information

locators that have their motions determined by a constraint. Although you can use the XMT 'exp' function with 'Parents of selected objects' selected, this export process is very slow.

IMPORTANT: If your locators can simply be parented to the objects that they should move with, then make sure to delete any constraint nodes that you used to position them before parenting.

IMPORTANT: It is recommended that you open the .csv output file to make sure that no locators have NaN for all frames.

Save the animation scene.

5. Calculate alpha shapes in Matlab

The script 'dynamicEndocastBeta.m' uses the locator coordinates, as well as a user-specified alpha value, to generate an alpha shape (aka alpha hull) for each frame. The alpha value is the maximum radius of curvature of the alpha shape, and it adjusts how tightly (or loosely) the alpha shape 'shrink wraps' the locator coordinates. A lower alpha value creates a more 'tightly wrapped' alpha shape than a higher alpha value. The alpha shapes are exported as .obj files. The script also calculates the volume of each alpha shape and exports a single .csv file that contains the alpha shape volume at each frame.

In Matlab, open and run the script 'dynamicEndocastBeta.m.' Five windows will pop up, followed by a plot of alpha shape volume over time.

First - Select the .csv file that contains the endocast locator coordinates.

Second - Select the folder where you want to save all the alpha shape (.obj) files. These files will be named automatically as 'Alpha_Hull_AV(alpha value)_(frame number)_frame' (e.g. 'Alpha_Hull_AV4_400_frame.obj').

Third - Enter the alpha value that you want to use. We recommend starting with an alpha value of 4 (and then adjusting the alpha value in future iterations as you improve the fit of your alpha shapes (Step 7).

Fourth - Enter a name (and location) for the .csv file that will contain the alpha shape volume at each frame. This file isn't necessary for any of the following steps, but may be useful for your analyses.

Fifth - This window will show the volumes of the alpha shapes in each frame. Look at this plot to get a sense of how volume is changing over time, and how noisy the volume data are, and if there are any discontinuities or outliers. Then close the plot.

You should now have a folder of .obj files (one alpha shape for each frame) and a single .csv file that contains the alpha shape volume at each frame. The units of these volume values will be the same as your XROMM animation. Usually this is cm (which means the volume data will be in cubic centimeters), but ultimately it will depend on the units of your calibration object.

Journal of Experimental Biology • Supplementary information

To see a pre-made set of alpha shapes (created from the example endocast locators provided in ‘Endocast_Tutorial_Ref_Scene.ma’) and their volumes at each frame, open the files ‘Endocast_Tutorial_Example_Alpha_Shapes.zip’ and ‘Endocast_Tutorial_Example_Volume_Data.’

6. **Import alpha shapes into Maya**

The script ‘impHulls’ imports the alpha shape .obj files and sets keyframes for each .obj so that each alpha shape is only visible for the frame that it is associated with. It groups (i.e. parents) the alpha shapes under a single node called ‘Hull: AlphaHulls.’ It also creates a locator called ‘HullVolume’ that has an attribute called ‘Volume,’ which contains that volume of the alpha shapes at each frame. Note: Currently you can only import one set of alpha shapes at a time. If you try to import a second set of obj files with the impHulls script, it will delete your first set and replace it with the second set.

Open the scene ‘Endocast_Tutorial_Animation.ma’ (the scene that you created, not the example animation scene that was provided) in Maya and enter ‘impHulls’ into the MEL command line (if this results in an error, check that the ‘impHulls.mel’ file is in the correct location on your computer, following the instructions at the beginning of this tutorial). In the first window that pops up, navigate to your folder of alpha shape .obj files, and select the ones you want to import. If you select a lot of files (a few hundred or more) this may take a while. For this tutorial, we recommend selecting the alpha shapes that correspond to frames 400-450. In the second window that pops up, enter the alpha value of the alpha shapes (as specified in their file names, e.g. “..._AV4_…”). This information is needed so that the alpha shape files can be read in.

You should now have a skeletal animation with both the bone models and the alpha shapes forming a dynamic endocast. Look through the animation and check for regions or frames during the behavior of interest when the alpha shape doesn’t fit well.

Tip: When selecting alpha shape .obj files to import, if you use the ‘shift’ button to select a range of files, we have observed that sometimes files in the middle are not selected, so you will be missing alpha shapes in certain frames in your Maya scene. When selecting files this way, be careful and make sure all files are selected.

Tip: Select the node ‘Hull: AlphaHulls’ and modify its visibility, color, etc. in order to change those properties for all of the alpha shapes.

Tip: Select the ‘Volume’ attribute of the locator ‘HullVolume’and display it in the Graph Editor to view the change in volume during the animation.

To see a file with Step 6 completed, open ‘Endocast_Tutorial_Example_Animation_Scene.ma,’ which contains alpha shapes created from the example endocast locators that were provided in ‘Endocast_Tutorial_Ref_Scene.ma.’

Journal of Experimental Biology • Supplementary information

Journal of Experimental Biology • Supplementary information

7. Improve the fit of your alpha shapes

If you observe that the the alpha shapes are severely penetrating the bones, not matching the internal surface of the bones, becoming concave as bones spread apart from each other, or not capturing the volume of interest in other ways, then it is necessary to go back and adjust the alpha value or adjust the endocast locators.

Some slight interpenetration is unavoidable and will have a small effect on volume. If it remains relatively consistent throughout the behavior, then it will have a minimal effect on volume change. The volume of an alpha shape at any single frame includes the volume of the fluid of interest, as well as the volume of soft tissue (and bone) that is within the bounds of the alpha shape. However, the dynamic endocast method is used to measure instantaneous volume change and assumes that the change in volume between frames is attributed to the change in volume of the fluid of interest (e.g. the water sucked inside a fish's mouth). This assumption is best met if bone and alpha shape interpenetration is consistent throughout the behavior.

To improve your endocast, you can either adjust the alpha value or the locator set. If the alpha shapes do not capture all of the volume of interest, it may be necessary to increase the number of locators or increase the alpha value. If the alpha shapes interpenetrate the bones, it may be necessary to decrease the alpha value or increase the number of locators. If there are problematic regions where the alpha shape does not capture the region of interest, it may be necessary to increase the number of locators or get creative with their placement.

7a. Adjusting the alpha value

Generating an alpha shape creates a 3D surface that encloses a set of 3D points, and the alpha value roughly controls how concave/convex the surface of the alpha shape is. A lower alpha value creates a more concave surface, while a higher alpha value creates a more convex surface.

In relation to the endocast method, a lower alpha value can cause the alpha shape to form more depressions, especially where (and when) the bones (and their attached locators) move furthest apart from each other, while increasing the alpha value can cause more bone interpenetration. When adjusting the alpha value, try to balance the trade-off between these effects.

To change the alpha value, rerun the script 'dynamicEndocastBeta.m' in MATLAB and enter a different alpha value when prompted. Then, repeat Steps 5 and 6 and see whether you are satisfied with the results. Since you cannot import multiple sets of alpha shapes into a single scene, you may want to save duplicate animated scenes: each with alpha shapes created from a different alpha value.

7b. Adjusting the endocast locators

Adding or moving locators can be an effective way to improve the fit of the alpha shapes in regions where they are not performing well. Depending on your dataset, you may have to get creative about how you place, parent, or constrain the locators. Below, are a few possibilities for placing locators, beyond simply parenting them to the bone meshes. These examples are

intended to give you potential options, but are not exhaustive. Creative solutions will look different in every dataset.

To edit endocast locators, open the reference scene in Maya and make changes (as described below). Then, open up an animation scene and check that the new locator set is properly animated. Once you have finished updating the positions of the locators in your reference scene, repeat steps 4, 5, and 6 and verify that the alpha shapes properly capture the cavity being studied.

7b. i. Extending the midsagittal plane

Issue:

In this tutorial, we use a midsagittal plane to divide the fish head in half to generate an endocast of oropharyngeal volume for the left side of the head. Locators were placed along the midsagittal plane and parented to the neurocranium. These locators needed to be placed along the plane such that they fit within the cross-section of the oropharyngeal cavity when the mouth is at its *smallest* resting position (otherwise they will create unrealistic endocast shapes that poke out of the oropharyngeal cavity when the mouth closes). However, when the oropharyngeal cavity is expanded, the cross-section of the oropharyngeal cavity grows but the locators on the midsagittal plane remain clustered in a small region (they don't move in relation to the neurocranium or each other). This can cause the alpha shapes to have depressions along the midsagittal plane in areas further away from the cluster of locators.

Solution:

You can fix this by opening the reference scene in Maya, placing additional locators along the midsagittal plane (that are still within the cross-section of the oropharyngeal cavity when the mouth is at its *smallest* resting position), and then parenting them to bones such as the cleithrum (which has a symphysis along the midsagittal plane). As the oropharyngeal cavity expands and its cross-section grows, these locators will move along the midsagittal plane and span the larger cross-section.

IMPORTANT: Check how the locators look in an animated Maya scene to make sure that your midsagittal locators don't move into weird, abnormal places as the bones expand and pull the locators along with them.

7b. ii. Bridging a gap: Point constraining a locator

Issue:

As bones move away from each other during a behavior, the alpha shapes can form depressions in the areas where the locators have become more spread out.

Solution:

You can fix this by creating a locator that will always be positioned halfway between two spots where the bones spread apart the most (e.g. the ventral tips of the cleithrum and ceratohyal). To do this:

Journal of Experimental Biology • Supplementary information

- Open the reference scene in Maya.
- Identify a gap between two bones that you want to bridge. If there aren't already locators there, then place a locator on each bone on either side of the gap (let's call them locators A and B). Now create a new locator (locator C).
- Point constrain locator C to locators A and B. Locator C should now be positioned halfway between locators A and B (and there should now be a constraint node parented under locator C – do not delete this constraint node)
    - If locator C does not move as expected, then make sure you selected locator C last.
    - If locator C still does not move as expected, then open up the settings for the point constraint (Constraint > click on the square to the right of 'Point'). Make sure that 'Maintain offset' is unchecked, that the offset is zero for all axes, and that 'Constrain axes' is set to 'all.' If these settings were not configured properly, then delete the constraint node that is parented to locator C and redo the point constraint.
- Locator C is not parented to anything, and instead its position is determined by the positions of the locators that it is constrained to.
- If one locator is not enough to bridge the gap between the bones, you can repeat this process with an additional locator. For example, you can create a locator D, and then point constrain it to locators A and C.

Open up the animation Maya scene and confirm that the locator(s) move dynamically with the bones.

IMPORTANT: When you export the locator coordinates, you have to use the 'expTransWSbyFrame.mel' script. If you use the XMT 'exp' function with 'Hierarchy of selected objects' selected in the 'Advanced Options,' the locators that are parented to the constrained cylinder will have NaN for all of their coordinates. Although you can use the XMT 'exp' function with 'Parents of selected objects' selected, this export process is very slow.

7b. iii. Bridging a gap: Parenting locators to a constrained object
Issue:
As bones move away from each other during a behavior, the alpha shapes can form depressions in the areas where the locators have become more spread out.

Solution:
You can fix this by parenting locators to an object that bridges a gap between two bones. Specifically, you can create a slender cylinder that has one end fixed to a spot on a bone (e.g. locator A from the example described above) and has its other end aimed at a spot on another bone (e.g. locator B from the example described above).

Journal of Experimental Biology • Supplementary information

This is a similar solution to the one described above, and may be more or less suitable to you, depending on your dataset. It is a bit more clunky, but we include it here because it illustrates how using an aim constraint can be helpful for placing locators creatively.

- Open the reference scene in Maya.
- Identify a gap between two bones that you want to bridge. If there aren't already locators there, then place a locator on each bone on either side of the gap (let's call them Locators A and B).
- Create a cylinder (Create > Polygon primitives > click the square to the right of 'Cylinder') with radius '0.05,' height '2.0,' height divisions '2,' and axis 'Z.'
- Move the cylinder's pivot to one end of the cylinder, such that it is centered on the end of the cylinder.
    - Specifically, move the pivot in the direction of negative translations along the Z axis. It will look like the blue Z-axis is pointing from one end of the cylinder towards the other.
- Point constrain the cylinder to Locator A.
- Aim constrain the cylinder to Locator B.
    - Select Locator B and then also select the cylinder.
    - Go to the options menu for the aim constraint (While in the 'Animation' menu bar: Constrain > Aim > click on the square to the right of 'Aim'). In the Aim Constraint Options window, set the 'Aim vector' to '0, 0, 1' and the 'Up vector' to '0, 1, 0.' Click Apply.
    - If the cylinder is aiming the wrong way by 180 degrees, then delete the aim constraint, move its pivot to its other end, and redo the constraints.
    - If the cylinder is aiming the wrong way by 90 degrees, then delete the aim constraint, and change the 'Aim vector' settings before re-applying the aim constraint.
- Create two endocast locators that move with the cylinder.
    - Create two locators.
    - Use the 'snap to vertex' tool to position them along the cylinder. Place one along the length of the cylinder, and place the second at the floating end.
    - Parent them to the cylinder.
- Duplicate the cylinder. Let's call it Cylinder B.
- Delete the constraint nodes that are parented under Cylinder B.
- Point constrain Cylinder B to Locator B and aim constrain it to Locator A, as described above.

Open up the animation Maya scene and confirm that the cylinders slide past each other as the bones spread apart.

IMPORTANT: Check that the cylinders aren't longer than the shortest distance between the bones (at any time during the behavior, nor in any other trial). If the cylinders are too long, then the cylinder and locators will cause the endocast to poke outside of the region

Journal of Experimental Biology • Supplementary information

of interest. If a cylinder isn't the right length, then you have to create a new cylinder, and change the 'height' in the options window.

IMPORTANT: DO NOT change the scale of either cylinder. If you do, the exported locator coordinates will be wrong.

IMPORTANT: When you export the locator coordinates, you have to use the 'expTransWSbyFrame.mel' script. If you use the XMT 'exp' function with 'Hierarchy of selected objects' selected in the 'Advanced Options,' the locators that are parented to the constrained cylinder will have NaN for all of their coordinates. Although you can use the XMT 'exp' function with 'Parents of selected objects' selected, this export process is very slow.

Journal of Experimental Biology • Supplementary information

Glossary of Maya hotkeys and tools

Parenting: select the 'child' object(s) (e.g. a locator), shift+select the 'parent' object, press 'p'
Causes the 'child' to inherit the transformations (rotation and translation) of the 'parent'.

Duplicating: select the object, press 'command' + 'd' (on mac) or 'control' + 'd' (on windows)
Creates a copy of the selected object and replicates the hierarchy position of the selected object.

Focusing: (optionally, select an object), press 'f' while mouse is hovering in the viewport
Centers the camera on the selected object, or on all objects in the scene if nothing is selected.

Setting a keyframe: select the object, confirm that you are at the desired frame, press 's'
Specifies (locks in) an object's position and attributes at a given point in time.

'Move' tool: press 'w'
Allows you to move objects, either along each of three axes or freely along all axes.

'Snap to vertex' tool: click on the object that you want to reposition (e.g. a locator), switch to the 'Move' tool, press and hold down 'v', then drag the object.
Restricts positioning of the object so it can only be placed along the vertices of visible meshes.

'Live object' tool: select the object, click on the 'make live' magnet icon (i.e. rightmost magnet icon in the Maya 'status line,' which is the row of icons that is usually found above the 'shelves'). Click on the 'make live' icon again to toggle the tool off.
Restricts positioning of other objects so they can only be placed along the live object's surface.

Point constraint: select the object(s) that will drive the motion, then shift+select the object that you want to constrain. While in the 'Animation' menu bar, click Constraint > Point. The last object selected will now be point constrained to the position of the other object(s).
Causes an object to move to and follow the position of another object, or the average position of several objects.

Aim constraint: select the object(s) that will drive the motion, then shift+select the object that you want to constrain. While in the 'Animation' menu bar: Constrain > Aim. The last object selected will now be aim constrained to the position of the other object(s).
For the aim constraints described in this tutorial, you will need to use certain settings. Go to the options menu for the aim constraint (while in the 'Animation' menu bar: Constrain > Aim > click on the square to the right of 'Aim'). Set the 'Aim vector' to '0, 0, 1' and 'Up vector' to '0, 1, 0.'
Causes an object to point towards another object, or the average position of several objects.

Moving an object's pivot: select the object, switch to the 'Move' tool, press 'd' ('edit pivot' hotkey), (optionally, press and hold down 'v' ('snap to vertex' hotkey)). Click and drag the center of the object's pivot to move it.
Allows you to move an object's pivot (center of rotation).

Journal of Experimental Biology • Supplementary information

References

Li, E. Y., Kaczmarek, E. B., Olsen, A. M., Brainerd, E. L. and Camp, A. L. (2022). Royal knifefish generate powerful suction feeding through large neurocranial elevation and high epaxial muscle power. *J. Exp. Biol.* **225**.

Kaczmarek, E. B., Li, E. Y., Capano, J. G., Falkingham, P. L., Gatesy, S. M., Brainerd, E. L., Camp, A. L. Precision and accuracy of the dynamic endocast method for measuring volume changes in XROMM studies.
